# Systematic Review and Meta-Analysis of Rituximab for Steroid-Dependent or Frequently Relapsing Nephrotic Syndrome in Children

**DOI:** 10.3389/fped.2021.626323

**Published:** 2021-07-22

**Authors:** Xia Gao, Yan Wang, Zichuan Xu, Huiying Deng, Huabin Yang, Fu Zhong

**Affiliations:** ^1^Nephrology Department, Guangzhou Women and Children's Medical Center, Guangzhou, China; ^2^Graduate School, Ningxia Medical University, Yinchuan, China; ^3^Neonatology Department, Northwest Women and Children's Hospital, Xi'an, China

**Keywords:** rituximab, meta-analysis, systematic review, effect and safety, nephrotic syndrome

## Abstract

**Objective:** To explore the effectiveness and safety of rituximab (RTX) for steroid-dependent or frequently relapsing nephrotic syndrome *via* a systematic review and meta-analysis.

**Methods:** All the literature about RTX therapy for childhood nephrotic syndrome (NS) on PubMed, Web of Science, Cochrane Library, EMBASE, and Chinese biomedical literature database published before November 1, 2019, were conducted and selected according to the preset criteria. The Cochrane bias risk assessment tool was used to evaluate the quality of the literature included. The outcome data were analyzed by RevMan 5.3 software.

**Results:** There were six RCT studies that met the inclusion criteria with a moderate quality after evaluation. At the end of the treatment, the relapse rate of NS in the RTX group reduced significantly when compared with that in the control group [odds ratio (OR) = 0.11, 95% confidence interval (CI) (0.03, 0.43), *p* = 0.001]. The number of patients in the RTX group used less steroid or/and calcineurin inhibitors significantly than that in the control group [OR = 0.05, 95% CI (0.01, 0.28), *p* = 0.0007]. For children who were steroid-dependent, RTX treatment significantly reduced the dosage of the steroid, compared with that in control [standardized mean difference (SMD) = −1.49, 95% CI (−2.00, −0.99), *p* < 0.00001]. There was no significant reduction in protein excretion between the two groups [SMD = −0.33, 95% CI (−0.71, 0.04), *p* = 0.08]. Fewer serious adverse reactions of RTX in the six studies were reported and most adverse events were mild.

**Conclusion:** RTX is effective and safe for children with steroid-dependent or frequently relapsing nephrotic syndrome.

**Systematic Review Registration:** Identifier: CRD 42020150933. https://www.crd.york.ac.uk/prospero/. This review has been registered to the PROSPERO on 27 Feb 2020.

## Introduction

The incidence of idiopathic nephrotic syndrome (NS) is 2–4 per 100,000 children (1). Prednisone is the core treatment for NS. There are still some patients who relapse when the steroid was withdrawn or tapering. According to their recurrence rate, these patients were classified as steroid-dependent nephrotic syndrome (SDNS) and frequently relapsing nephrotic syndrome (FRNS) (2, 3). The long-term use of steroids in children could result in serious adverse effects such as growth retardation, obesity, osteopenia, hypertension, and cataract (4). The SDNS or FRNS required a combined therapy of steroid and immunosuppressive agents, such as cyclophosphamide (5), cyclosporine A (CSA) (6), tacrolimus (TAC) (7), mycophenolate esters (8), and vincristine (VCR) (9). These immune suppressants brought the patients a higher remission rate, together with even more serious adverse reactions, such as renal toxicity, hyperglycemia, headache, and dyslipidemia (10).

Rituximab (RTX), a monoclonal antibody targeting the CD20 antigen of B lymphocytes, was first proposed to lymphoma (11) and rheumatoid arthritis (12). Recently, RTX was introduced to the relapsing NS (13–15) and brought the patients a long-lasting remission. Other findings suggested that RTX could also bring better benefits to children. In Guigonis's clinical trials (16), 22 patients were given RTX treatment. Nineteen children received remission. Additionally, there were some other case reports that showed that RTX induced remission in patients (17–20), whereas some studies reported no benefits of RTX to patients that were resistant to steroids and CNIs. Kari et al. (21) observed that in four children with steroid-resistant NS, after RTX protocol, only one child received a short remission and then relapsed, and the other three children showed little effects.

We aimed to evaluate the efficacy of RTX for SDNS or FRNS in children compared to the conventional treatment by meta-analysis and to summarize the adverse effects to evaluate the safety. The research was introduced as follows.

## Methods

### Electronic Searches

A unified search strategy was adopted for the selected five databases to ensure consistency. The medical subject heading of the disease was restricted to Nephrotic Syndrome, the text words were Nephrotic Syndromes; Syndrome, Nephrotic; and Syndromes, Nephrotic; the medical subject heading of the intervention was RTX, and the text words were CD20 Antibody, Rituximab; Rituximab CD20 Antibody; Mabthera; IDEC-C2B8 Antibody IDEC C2B8 Antibody; IDEC-C2B8; IDEC C2B8; GP2013; and Rituxan. The subject heading of study type was Randomized Controlled Trial, and the text words were RCT; Random. Boolean operations were used to connect these subject words and text words to form search formulas. Two researchers checked the search results multiple times after independent searches and saved the search results in EndNote software.

### Study Selection Criteria

The study type was restricted to the randomized controlled trial. We selected children with SDNS or FRNS who were younger than 18 years old. Two groups of patients were studied; one received any dose of RTX. The other was considered as the control, receiving conventional drugs, such as steroid and TAC/CSA. Since the use of RTX is only intended for non-genetic nephrotic syndrome, genetic nephrotic syndrome is not included in this study.

The main outcome was the relapse number. The secondary outcomes were the number of using steroids or/and calcineurin inhibitors, the dose of steroids, and the degree of proteinuria.

### Literature Bias Evaluation

The risk of bias was evaluated by the Cochrane bias risk assessment tool of RevMan software. The risk of bias included selective bias, performance bias, detection bias, attrition bias, reporting bias, and other biases.

### Data Extraction

Two researchers extracted relevant data to a pre-designed form independently. The disputed data were discussed with a third researcher. The extracted information included the following sections: (1) literature basic information, (2) baseline data, (3) interventions, and (4) outcomes. The extracted data were mostly represented by the mean and standard deviation (SD).

### Data Analysis

The data analysis was performed by RevMan (version 5.3.5). For dichotomous variables, the odds ratio (OR) and 95% confidence interval (CI) were calculated. Continuous variables were analyzed by the standardized mean difference (SMD) and 95% CI. The *Q*-test and *I*^2^ analysis were used to test the heterogeneity of the literature. When *p* < 0.10 in the *Q*-test or *I*^2^ > 50% in *I*^2^ analysis, the random-effect model was used; otherwise, the fixed-effect model was used. We then attempted to determine the source of heterogeneity, and subgroup analysis was continued if possible. *p* < 0.05 denotes that the difference was considered significant.

## Results

**Figure 1:**
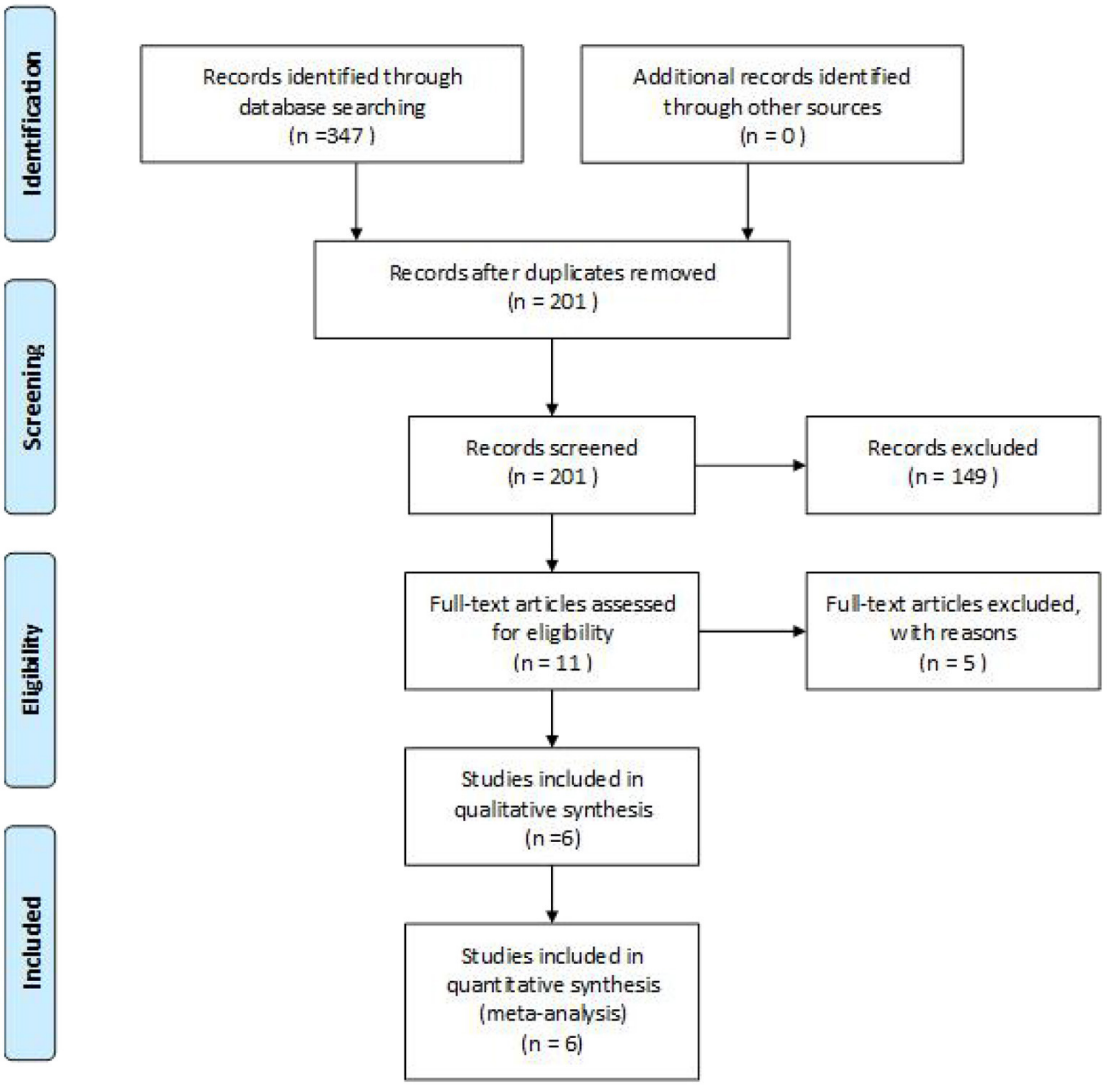
The search results and selection details.

A total of 347 eligible articles were retrieved, namely, 42 from PubMed, 103 from Web of Science, 67 from the Cochrane Library, 103 from EMBASE, and 32 from the Chinese Biomedical Literature Database. A total of 146 duplicate articles were eliminated by Endnote software. After selecting the title and abstract, 190 articles were excluded. The remaining 11 articles were selected for further reading. Among them, studies from Webb et al. (22), Deble-Bertin et al. (23), and Sinha et al. (24) were excluded because these were cohort studies. Research from Basu et al. (25) was excluded due to the addition of TAC in the control group. The study of Kamei et al. (26) was a long-term follow-up observational study, which was excluded due to the lack of a control group. Finally, six articles (27–32) were included in our study, and the screening flow chart is shown in Figure 1. The general data of the study design is shown in Table 1, and the baseline data of the study patients are shown in Table 2.

**Table 1:** General information of the included studies.

**References**	**Country**	**Histology RTX group**	**Histology con group**	**Treatment RTX group**	**Treatment con group**	**Follow-up**
Ahn et al. (27)	Korea	MCD: 23FSGS: 2Unknown: 10	MCD: 8 FSGS: 1 Unknown: 7	RTX: a single dose of intravenous RTX (375 mg/m^2^; maximum of 50 0 mg); PED; CNI	PED (60 mg/m^2^/day, tapered by 25% every 4 weeks); CNI	12 M
Boumediene et al. (29)	France	MCD: 9Unknown: 1	MCD: 11 FSGS: 1 Unknown: 1	RTX: two infusions at 1-week interval of Rituximab (375 mg/m^2^); MMF: CNI; PED	PED (decreased every 2 weeks by 25%); MMF; CNI	6 M
Iijima et al. (28)	Japan	MCD: 21FSGS: 2Unknown: 1	MCD: 23 FSGS: 1	RTX: once weekly for 4 weeks (375 mg/m^2^; maximum of 500 mg); MP; chlorpheniramine maleate; paracetamol	PED (60 mg/m^2^/day, maximum of 80 mg/day, decreased every 2 weeks by 50%)	12 M
Magnasco et al. (30)	Italy	MCD: 3FSGS: 10Unknown: 2	MCD: 4 FSGS: 9 Unknown: 3	RTX: 375 mg/m^2^, given intravenously twice (at randomization and after 2 weeks); PED; MP; CNI; chlorpheniramine maleate; paracetamol; ARB; ACEI	PED (median 0.42 mg/kg/day, after 30 days decreased every week by 0.3 mg/kg); CNI; ARB; ACEI	18 M
Ravani et al. (31)	Italy	MCD: 13FSGS: 7Unknown: 7	MCD: 6 FSGS: 10 Unknown: 116	Rituximab (375 mg/m^2^) was given intravenously once or twice; ARB; MP; PED; CNI; ACEI; chlorpheniramine maleate; paracetamol	PED (tapered off by 0.3 mg/kg per week if proteinuria was <1 g/day); CNI; ACEI; ARB	12 M
Ravani et al. (32)	Italy	Unknown: 15	Unknown: 15	Rituximab (MabThera/RITUXAN;375 mg/m^2^); PED; MP; chlorpheniramine maleate; paracetamol; ACEI; ARB; CNI; CYC	PED (tapered off by 0.3 mg/kg per week starting at 30 days); ACEI; ARB; CNI; CYC	12 M

*MCD, minimal-change disease; FSGS, focal and segmental glomerular sclerosis; PED, prednisone; CNI, calcineurin inhibitors (cyclosporine or tacrolimus); MMF, mycophenolate mofetil; MP, methyl prednisone; ARB, angiotensin receptor inhibitor; ACEI, angiotensin-converting enzyme inhibitor; CYC, cyclophosphamide*.

**Table 2:** Basic characteristics of included studies.

	**Group**	**Ahn et al. (27)**	**Boumediene et al. (29)**	**Iijima et al. (28)**	**Magnasco et al. (30)**	**Ravani et al. (31)**	**Ravani et al. (32)**
Sex (M/F)	RTX group	26/9	10/0	18/6	10/6	24/3	10/5
	Con group	13/3	6/7	16/8	9/6	19/8	11/4
Age (years)	RTX group	13.5 ± 5.0	11.1 ± 1.2	11.5 ± 5.0	8.5 ± 4.4	10.2 ± 4.0	6.9 ± 3.6
	Con group	12.5 ± 4.2	12.3 ± 1.0	13.6 ± 6.9	7.3 ± 3.7	11.3 ± 4.3	6.9 ± 3.1
Weight (kg)	RTX group	-	-	44.0 ± 18.6	31 ± 17	39.6 ± 15.2	30.0 ± 16
	Con group	-	-	47.5 ± 15.6	30 ± 17	45.5 ± 19.3	31.0 ± 41.0
Duration of NS (years)	RTX group	8.7 ± 4.9	-	7.9 ± 4.7	2.5 ± 8.4	5.7 ± 3.5	2.7 ± 2.4
	Con group	7.4 ± 4.9	-	8 ± 5.4	1.4 ± 5.6	7.8 ± 4.0	2.0 ± 2.5
Serum creatinine (μmol/L)	RTX group	-	-	39.8 ± 13.3	46.9 ± 30.9	48.6 ± 26.5	35.4 ± 17.7
	Con group	-	-	44.2 ± 15.9	48.6 ± 36.2	48.6 ± 17.7	36.2 ± 8.8
Serum albumin (g/dl)	RTX group	-	-	3.4 ± 0.6	2.4 ± 0.6	3.6 ± 0.9	3.8 ± 0.3
	Con group	-	-	3.4 ± 0.5	2.3 ± 0.5	3.2 ± 0.8	4.0 ± 0.4
Dose of prednisone (mg/kg/day)	RTX group	0.5 ± 0.4	-	-	0.5 ± 1.4	0.6 ± 0.4	0.6 ± 0.4
	Con group	0.3 ± 0.3	-	-	0.4 ± 0.8	0.6 ± 0.5	0.6 ± 0.5
CYC/TAC	RTX group	35/15	5/4	16/0	8/8	19/8	-
	Con group	16/6	6/3	16/0	7/8	19/8	-

*CYC, cyclophosphamide; TAC, tacrolimus*.

**Figure 2:**
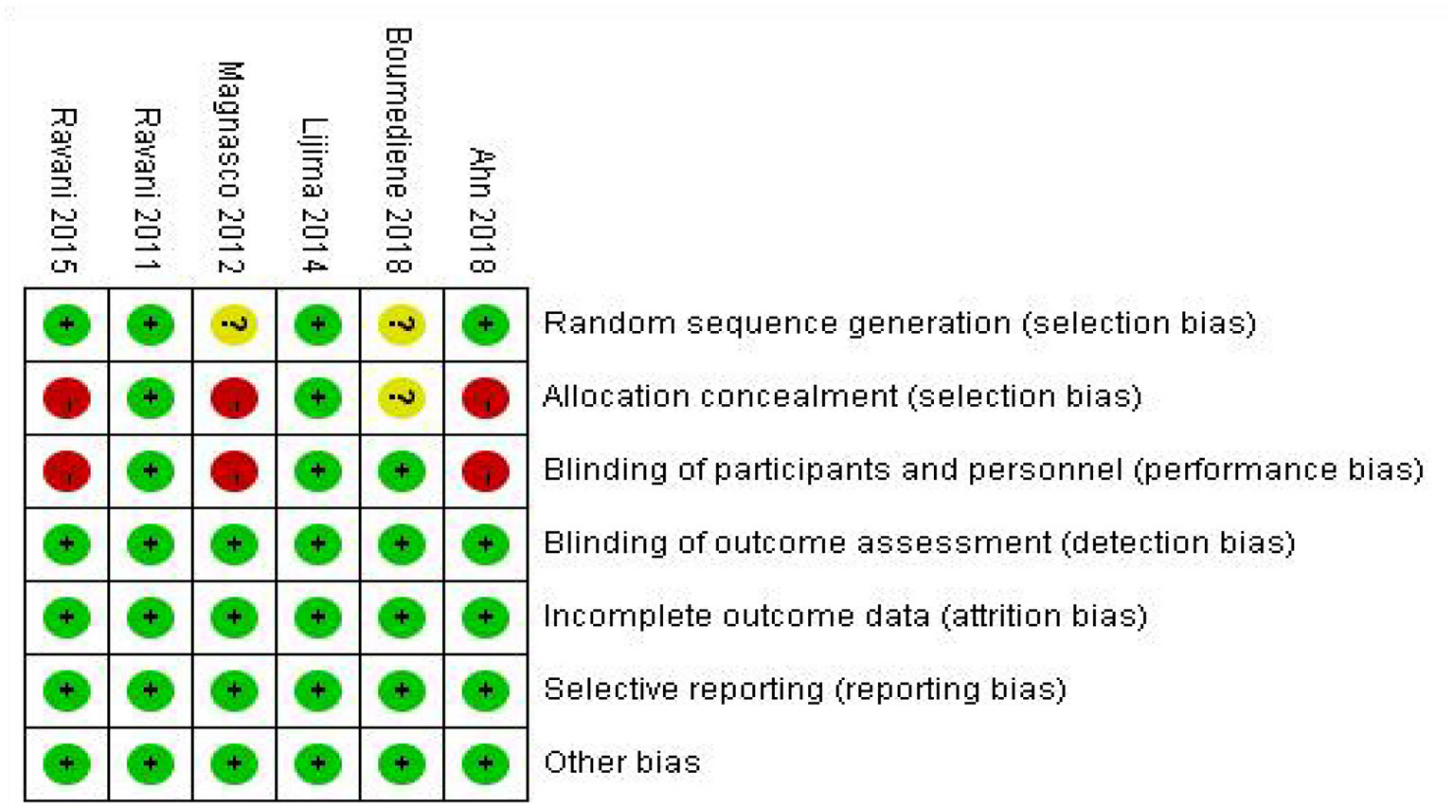
Risk of bias summary.

After repeatedly reading the six articles, the Cochrane Bias Risk Assessment Tool was used to evaluate the RevMan software. The results of the included literature bias assessment are shown in Figure 2.

### The Relapse Numbers

**Figure 3:**
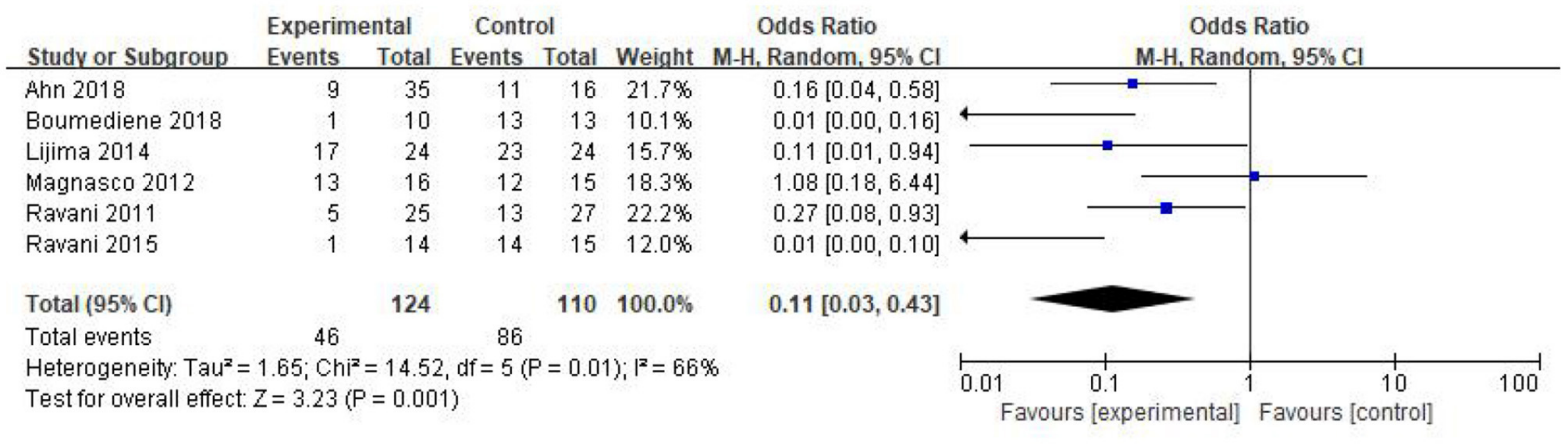
Forest plot showing a meta-analysis for the RTX group vs. control group on the relapse numbers.

In this study, six studies reported the relapse number of NS in the RTX group and the control after the corresponding treatment. A total of 46 patients of 124 in the RTX group and 86 patients of 110 in the control relapsed, separately. RTX could significantly reduce the NS relapse during the observation period [OR 0.11, 95% CI (0.03, 0.43), *p* = 0.001]. The random-effect model was selected due to the large heterogeneity in this analysis (*I*^2^ = 66%, *p* = 0.01), and the results are shown in Figure 3.

### The Number of Patients Using Steroids or/and CNI

**Figure 4:**
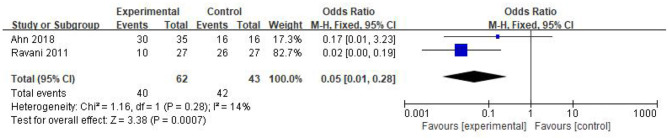
Forest plot showing a meta-analysis for the RTX group vs. control group on the number of patients using steroids or/and CNI.

In this study, there were two studies documenting the number of patients using steroids or/and CNI after the corresponding treatment. Twenty patients of 62 in the RTX group discontinued steroids or/and CNI and achieved remission. Only 1 patient of 43 in the control withdrew steroids or/and CNI. Thus, we concluded that RTX holds great potential of reducing steroids or/and CNI treatment [OR 0.05, 95% CI (0.01, 0.28), *p* = 0.0007]. The fixed-effect model was used according to the heterogeneity, which was acceptable (*I*^2^ = 14%, *p* = 0.28); the results are shown in Figure 4.

### Steroid Dosage

**Figure 5:**
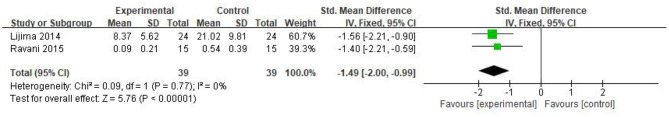
Forest plot showing a meta-analysis for the RTX group vs. control group on the steroid dosage.

Three studies recorded the steroid dosage after the corresponding treatment. Among them, the research from Ahn et al. (27) was excluded because of the large heterogeneity. The two remaining articles were included for further analyses and a total of 78 patients were studied, 39 in the RTX group and 39 in the control. The SMD was used for analysis due to the different units of steroids in the two research, which showed that the steroid dosage was significantly reduced after RTX treatment [SMD −1.49, 95% CI (−2.00, −0.99), *p* < 0.00001]. There was no obvious heterogeneity (*I*^2^ = 0%, *p* = 0.77) and the fixed-effect model was used; the results are shown in Figure 5.

### Proteinuria Excretion

**Figure 6:**
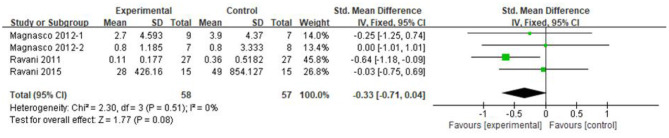
Forest plot showing a meta-analysis for the RTX group vs. control group on the proteinuria excretion.

Three articles recorded urea protein excretion in 115 patients, of whom 58 were in the RTX group and 57 were in the control group. Because the units of proteinuria were not uniform, the SMD was used for analysis. Compared with that in the control group, the proteinuria excretion in both RTX and control decreased, and the difference was not statistically significant [SMD −0.33, 95% CI (−0.71, 0.04), *p* = 0.08], as shown in the forest plot (Figure 6).

### Safety

Adverse events, such as late-onset neutropenia, hypogammaglobulinemia, and increased risk of infections, have been rarely reported. Slight adverse reactions and infusion reactions were found in all included studies, which could be alleviated by reducing the speed of drug infusion or providing supportive treatment.

### Publication Bias

In the main outcome of the relapse number, we used Stata software to evaluate publication bias. The results were as follows: *p* = 0.060 in the Begg test, *p* = 0.022 in the Egger test.

## Discussion

We included six randomized controlled trials. A total of 234 children were included and randomly assigned to the RTX group (124 children) and the control group (110 children). We found that when compared to that in control, RTX could reduce the relapse number of NS, the use of steroids or/and calcineurin inhibitors, and the dosage of steroids, while RTX could not decrease the protein excretion. There were fewer reports of serious adverse reactions of RTX during the therapy, and most adverse events were mild and could be alleviated after stopping RTX or reducing the infusion speed.

As known, the immune disorder was an extremely important pathogenic factor for relapsing nephrotic syndrome (33). The T cell developed dysfunction and secreted some chemical mediators. These abnormal cytokines could change and damage the glomerular filtration membrane. The interest in B cell as a potential pathogenic factor for nephrotic syndrome resulted in reorganization in recent years. The B cells could express co-stimulatory molecules and cytokines, which induced activation and the dysfunction of T cell. RTX induced apoptosis and depletion of B cell, thus inhibiting the interaction between B cells and T cells and reducing the recurrence of nephrotic syndrome in children. Additionally, some research found that RTX had a protective effect on podocytes in a non-immune pathway (34). RTX could bind to the sphingomyelin phosphodiesterase acid-like 3b protein (SMPDL3b) in lipid rafts expressed on podocytes. The combination of RTX and SMPDL3b played a role in protecting the structure and function of podocytes because SMPDL3b was a key protein that regulated the cytoskeleton of the podocytes.

The research found that RTX holds great potential in reducing the relapses of NS compared with control. Kemper et al. (35) conducted a multicenter retrospective clinical trial and found that 69% of patients achieved long-term remission and 48% of patients stopped immune-suppressant treatments. Sun et al. (13) reported that RTX treatment showed an effective rate of 91.67%, and the number of relapses was significantly reduced (*p* < 0.001). Sellier-Leclerc et al. (19) showed that only 23% of the children relapsed during the treatment of RTX. After long-term observation, the number of relapses was only 37%.

We found a big heterogeneity in the number of relapses in the meta-analysis, with *I*^2^ = 66%, *p* = 0.01. We presumed that the different statistical time cutoffs could be the source of heterogeneity and performed a subgroup analysis by the different cutoffs, 3, 6, and 12 months. We did get a negative result; thus, considering the different cutoff could not be the source of the heterogeneity. We found another interesting difference among the studies included in the mate analysis; the dose of RTX was different. We hypothesized the dose of RTX as the variant and performed subgroup analysis, but could not find the sources of the heterogeneity, either. Last, we considered that the heterogeneity of the analysis would be caused by different experimental design methods, small sample sizes, and different outcome indicators.

Two articles in this study documented the number of patients who took steroids or/and CNI. The mate analysis showed a significant decrease in patients taking steroids or/and CNI after RTX treatment. In the research of Ito et al. (36), 41 of 53 (77%) SDNS/FRNS patients discontinued steroid successfully. Of 53 SDNS/FRNS patients, 17 (31%) stopped CSA. Guigonis et al. (16) found that 19 patients (85%) discontinued treatment with one or more immunosuppressive agents, with no recurrence and no additional immunosuppressive agents. These results indicated that RTX treatment could reduce the usage and the dosage of steroids and other immunosuppressants.

Three articles reported the dosage of steroids after the RTX therapy. In the research of Ahn et al. (27), a total of 61 patients were enrolled, 40 in the RTX group and 21 in the control, and the ratio was 2:1, which resulted in a huge heterogeneity, and the research was excluded. The heterogeneity could be reduced from 55 to 0%. The fixed-effect model was used to analyze the two remaining articles and the results showed the significantly reduced dosage of the steroid after RTX treatment. Sun et al. (13) recorded less steroid usage (*p* = 0.014) and less relapse (*p* < 0.001) in 12 children in the next 6 months after RTX treatment. Gulati et al. (37) studied 57 children, 12 of whom could discontinue one or more immunosuppressive agents. In the other eight patients, the dose of prednisone could be gradually reduced to 0.3–0.5 mg/kg every other day. Ahn et al. (27) and Iijima et al. (28) reported that the average days without steroid application were 141 and 211 days, respectively, and the toxic and side effects of long-term use of steroids for children were avoided.

As known, reducing proteinuria played a key role in NS treatments. Hofstra et al. (38) also found that proteinuria decreased significantly (2–3 g/day) within 2 weeks. Our analysis did not find a significant difference in the reduced proteinuria levels after RTX therapy. The following factors could be responsible for the result. First, the number of patients enrolled in the research was so limited and we needed a larger sample size for the evaluation of the effect of RTX. Secondly, there were big heterogeneities among the patients. Besides the differences in pathological types, children with early drug resistance and late drug resistance were enrolled in the research of Magnasco et al. (30). Last, the proteinuria was affected by a variety of factors. In the study of Dahan et al. (39), the serum albumin level can affect proteinuria.

Most patients tolerated the RTX treatment well. A review from Bonanni et al. (40) reported that the most common adverse event was rash, dyspnea, fever, cough, and infusion-related itching. All these adverse reactions could be well-controlled. Recently, anti-CD20 antibodies from human beings such as Ofatumumab were undergoing clinical research (41–43) and the adverse events remained to be further evaluated.

All the literature in our research showed good quality and held less bias in the Cochrane bias risk assessment. Stata software was applied to assess publication bias, and *p* = 0.060 in the Begg test and *p* = 0.022 in the Egger test. The Egger test result with a high *p* = 0.022 indicates that there was a publication bias, and the small size of the sample could be accounted for it.

There are some limitations. First, the length of remission before the relapse could be considered as an indicator of the drug efficacy. Second, the follow-up time of included studies was inconsistent and all were short. Third, the characteristics of baseline are different, and the age of the two studies (30, 32) is younger than other studies. Finally, RTX therapy has a higher cost than steroid and CNI treatment, and the economic benefits of RTX needed further evaluation. So, we needed much larger, long-term, comprehensive, and controlled studies to further evaluate the clinical value of RTX.

## Data Availability Statement

The original contributions presented in the study are included in the article/supplementary material, further inquiries can be directed to the corresponding authors.

## Author Contributions

XG and FZ: guarantor of integrity of entire study and manuscript final version approval. XG and YW: study concepts. YW: study design, statistical analysis, manuscript definition of intellectual content, and manuscript revision/review. HD: literature research. ZX: data acquisition and data analysis/interpretation. HY: manuscript preparation and manuscript editing. All authors contributed to the article and approved the submitted version.

## Conflict of Interest

The authors declare that the research was conducted in the absence of any commercial or financial relationships that could be construed as a potential conflict of interest.
